# Endophytic bacterial communities in wild rice (*Oryza officinalis*) and their plant growth-promoting effects on perennial rice

**DOI:** 10.3389/fpls.2023.1184489

**Published:** 2023-08-14

**Authors:** Qinglin Tian, Yurui Gong, Shuang Liu, Menglin Ji, Rui Tang, Deting Kong, Zhifeng Xue, Linglin Wang, Fengyi Hu, Liyu Huang, Shiwen Qin

**Affiliations:** Key Laboratory of Biology and Germplasm Innovation of Perennial Rice From Ministry of Agriculture and Rural Affairs, School of Agriculture, Yunnan University, Kunming, Yunnan, China

**Keywords:** *Oryza officinalis*, endophytic bacteria, plant growth-promoting trait, perennial rice, biofertilizer

## Abstract

Endophytic bacterial microbiomes of plants contribute to the physiological health of the host and its adaptive evolution and stress tolerance. Wild rice possesses enriched endophytic bacteria diversity, which is a potential resource for sustainable agriculture. *Oryza officinalis* is a unique perennial wild rice species in China with rich genetic resources. However, endophytic bacterial communities of this species and their plant growth-promoting (PGP) traits remain largely unknown. In this study, endophytic bacteria in the root, stem, and leaf tissues of *O. officinalis* were characterized using 16S rRNA gene Illumina sequencing. Culturable bacterial endophytes were also isolated from *O*. *officinalis* tissues and characterized for their PGP traits. The microbiome analysis showed a more complex structure and powerful function of the endophytic bacterial community in roots compared with those in other tissue compartments. Each compartment had its specific endophytic bacterial biomarkers, including *Desulfomonile* and *Ruminiclostridium* for roots; *Lactobacillus, Acinetobacter, Cutibacterium* and *Dechloromonas* for stems; and *Stenotrophomonas*, *Chryseobacterium*, *Achromobacter* and *Methylobacterium* for leaves. A total of 96 endophytic bacterial strains with PGP traits of phosphate solubilization, potassium release, nitrogen fixation, 1-aminocyclopropane-1-carboxylate (ACC) deaminase secretion, and siderophore or indole-3-acetic acid (IAA) production were isolated from *O. officinalis*. Among them, 11 strains identified as *Enterobacter mori*, *E*. *ludwigii*, *E*. *cloacae*, *Bacillus amyloliquefaciens*, *B*. *siamensis*, *Pseudomonas rhodesiae* and *Kosakonia oryzae* were selected for inoculation of perennial rice based on their IAA production traits. These strains showed promising PGP effects on perennial rice seedlings. They promoted plants to form a strong root system, stimulate biomass accumulation, and increase chlorophyll content and nitrogen uptake, which could fulfil the ecologically sustainable cultivation model of perennial rice. These results provide insights into the bacterial endosphere of *O. officinalis* and its application potential in perennial rice. There is the prospect of mining beneficial endophytic bacteria from wild rice species, which could rewild the microbiome of cultivated rice varieties and promote their growth.

## Introduction

*Oryza officinalis*, with a diploid CC genome, is one of the native wild rice resources in South China. It has a suite of desirable agronomic traits similar to those of C4 plants, e.g., strong growth potential, high photosynthetic efficiency and large biomass accumulation (Kiran et al., 2013). This species exhibits both high tolerance to abiotic stresses (Hirabayashi et al., 2015; Liu et al., 2015; Kitazumi et al., 2018) and strong resistance to biotic stresses (Velusamy et al., 1990; Devanna et al., 2014; Zhang et al., 2014; Chen et al., 2022). Genetic resources of agronomic traits and resistance in *O. officinalis* have been utilized successfully for cultivated rice variety breeding and improvement.

In addition to their hereditary potential, wild plants utilize the plant-associated microbiome to expand their environmental adaptability, nutrient utilization efficiency and tolerance to biotic and abiotic stresses (Cordovez et al., 2019; White et al., 2019; Liu et al., 2022a). The main members of the phytomicrobiome originate from the endosphere, phyllosphere and rhizosphere (Singh et al., 2020). Despite the different microbial communities, plant plays the main role in the plant holobiont and determine the composition and function of the microbiome (Sanchez-Canizares et al., 2017). Previous studies have demonstrated that host genotype and phenotype have an active impact on microbiome assembly (Bulgarelli et al., 2015; Perez-Jaramillo et al., 2017; Zhang et al., 2019). Thus, wild plants harbour a higher abundance and more diverse microbiome than their domesticated cultivars (Zachow et al., 2014; Mommer et al., 2018). *O. officinalis* may harbour a largely unexplored functional microbiome, which will offer an understanding of its synergetic adaptive evolution and reinstate the microbiome diversity of cultivated rice that has been reduced through domestication.

Endophytes, as nonpathogenic microorganisms, are pivotal components of plant-associated microbiomes that colonize every accessible host plant tissue (Wani et al., 2015). Among them, endophytic bacteria are attracting much attention for their direct or indirect positive effects on plant development and health (Afzal et al., 2019; White et al., 2019). Endophytic bacteria have PGP mechanisms similar to those of rhizobacteria, and both are recognized as plant growth promoting bacteria (PGPB) (Afzal et al., 2019). Most PGPB convert macronutrients (potassium, phosphorous and zinc) in soil and atmospheric nitrogen into available nutrients for plant uptake. A large number of PGPB synthesize phytohormones to regulate plant metabolism and nutrient accumulation, e.g., indole-3-acetic acid (IAA), gibberellins and ethylene. Some PGPB release 1-aminocyclopropane-1-carboxylate (ACC) deaminase to lower endogenous ethylene levels and promote plant growth under abiotic stress. Moreover, some of them also generate volatile organic compounds (VOCs) to interfere with quorum sensing of phytopathogens, synthesize siderophores for iron competition with phytopathogens, produce hydrolytic enzymes and antibiotics to kill phytopathogens, and induce systematic-acquired resistance (SAR) to strengthen the host immune system. Furthermore, some PGPB are able to produce antiherbivore compounds (e.g., alkaloids) and toxins to deter feeding by insects and other herbivores (Wani et al., 2015; White et al., 2019; Kumar and Nautiyal, 2022). Some endophytic bacteria also protect their host from metal toxicities and remediate metal-contaminated fields (Franco-Franklin et al., 2021). Due to their effective endophytic colonization, endophytic bacteria usually expand more significant PGP effects on plants than rhizobacteria, especially under stress conditions (Chanway et al., 2000; Afzal et al., 2019).

Most endophytic bacteria have a wide host range. When isolated and inoculated into host plants or nonhost plants, many of them have been shown to promote plant health, enhance crop productivity and improve stress responses (Sun et al., 2016; Lopes et al., 2018; Wang et al., 2019; Wang et al., 2020b). Due to their environmentally friendly and sustainable nature, endophytic bacteria can be exploited as biofertilizers, biocontrol and bioremediation agents to improve agricultural management and add value to the agricultural market.

Perennial rice is a new type of rice variety with the capacity to survive and be harvested for consecutive years (Yao et al., 2022; Zhang et al., 2022). Perennial rice production saves seed and labour costs by eliminating sowing or planting after the first growth seasons. It has extended application in China, South and Southeast Asia and Africa (Zhang et al., 2017; Huang et al., 2018). Due to long-term cultivation, ecological intensification strategies should be adopted for perennial rice to maintain sustainable production and soil health. Microbial fertilizers are a multi-win solution for perennial rice management without the environmental and food safety concerns caused by chemical products. During continuous cultivation, microbial fertilizers may autonomously and continuously remedy the loss of host-associated microbes for perennial rice. However, there are no effective microbial fertilizers specifically for perennial rice. Therefore, endophytic bacterial composition and diversity in the root, stem and leaf tissues of *O. officinalis* were analysed using 16S rRNA gene Illumina sequencing technology in this study. Endophytic bacteria of *O. officinalis* were subsequently isolated and identified. Moreover, their PGP traits *in vitro* were screened and PGP effects on perennial rice were also investigated. Our results provide a basic understanding of the endophytic micro-ecosystem in *O. officinalis*. Endophytic bacterial strain resources collected in this study can be developed as biofertilizers in compliance with the simplified and sustainable cultivation model of perennial rice.

## Materials and methods

### Sample collection and processing

In April 2020, germinated seeds of *O. officinalis* were grown for 20 days in a tray with sterilized soil and three healthy seedlings were transplanted equidistantly into a pot (15 cm in length, 15 cm in width and 30 cm in height) filled with 3 kg of sterilized field soil. Seedlings of *O. officinalis* were planted in six pots with four replicates, and a total of 18 plants were used for sampling. The pots were maintained under controlled conditions with 14 h daylight at 28°C and a 10 h dark period at 25°C in the greenhouse of Yunnan University (Kunming city, Yunnan Province, 24° 82′ 76″ N, 102° 84′ 52″ E). They were well watered with tap water to ensure plant health and maintain the soil under flooded conditions. The roots, stems, and leaves from 6 individuals at the flowering stage were sampled as one replicate, and 3 replicates were used for further study. Samples were washed successively in 70% ethanol for 2~5 min and 2.5% sodium hypochlorite for 2~3 min to remove microorganisms on the surface of tissues. Finally, they were rinsed with sterile water 1~3 times, and the residual water was absorbed from the tissue surface with sterile filter paper (Zhang et al., 2013). The final rinse water was cultured on Luria broth (LB) medium plates to verify the surface sterilization effect. One batch of samples was flash frozen in liquid nitrogen and stored at -80°C for DNA extraction, and the other batch was prepared for the isolation of endophytic bacteria.

### 16S rRNA gene Illumina sequencing

DNA was extracted from tissue samples of *O. officinalis* using the OMEGA Soil DNA Kit (Omega Bio-Tek, Norcross, GA, USA); quantity and quality were measured using a NanoDrop NC2000 spectrophotometer (Thermo Fisher Scientific, Waltham, MA, USA) and agarose gel electrophoresis, respectively. PCR amplification of the V5-V7 region was performed using the forwards primer 799F (5’- AACMGGATTAGATACCCKG-3’) and the reverse primer 1193R (5’- ACGTCATCCCCACCTTCC -3’). PCR amplicons were purified with Vazyme VAHTSTM DNA Clean Beads (Vazyme, Nanjing, China) and quantified using the Quant-iT PicoGreen dsDNA Assay Kit (Invitrogen, Carlsbad, CA, USA). Paired-end sequencing was performed using the Illumina NovaSeq-PE250 platform at Shanghai Personal Biotechnology Co., Ltd. (Shanghai, China).

### Microbiome bioinformatics and statistical analysis

Raw sequences were processed to demultiplex, quality filter, denoise, merge, remove chimaeras and taxonomically annotate using QIIME2 (version 2019.4) (Bolyen et al., 2019). All raw sequences are publicly accessible in the Genome Sequence Archive (GSA) under accession number CRA008714 (https://ngdc.cncb.ac.cn/gsa/browse/CRA008714). After removing the sequences related to chloroplasts or mitochondria, amplicon sequence variants (ASVs) were generated for further analysis using the QIIME2 and R packages (version 3.2.0). Biomarkers were predicted with random forest classification. Alpha diversity (Shannon index, Chao1, Pielou index, Faith-PD, observed species and Good’s coverage) was analysed based on the normalized ASVs table and Wilcoxon rank-sum test. Beta diversity was visualized via nonmetric multidimensional scaling (NMDS) based on Bray-Curtis distance and ANOSIM similarity tests. Metacyc pathway analysis was conducted by the PICRUSt2 package (Douglas et al., 2020). Co-occurrence networks were constructed using SparCC and visualized using the R packages igraph and ggraph.

### Isolation and characterization of endophytic bacteria with PGP traits *in vitro*

Surface-sterilized tissue samples were ground gently in a mortar using sterile distilled water. Tenfold serial dilutions (10^-1^-10^-5^) of ground suspension (100 μL) were plated on nutrient agar (NA) medium. After 1-7 d of inoculation at 30°C, morphologically unique bacterial colonies were selected and purified on fresh NA plates by repeated streaking.

The PGP traits of inorganic phosphate solubilization, organic phosphate solubilization, potassium release, siderophore production and nitrogen fixation were determined on Pikovskaya’s agar medium (Pikovskaya, 1948), Mongina organophosphorus culture medium (Hu et al., 2009), potassium bacteria agar medium (Yaghoubi et al., 2021), chrome azurol S (CAS) agar medium (Balasjin et al., 2022) and nitrogen-free Ashby’s medium (Patil et al., 2010), respectively. The presence of a transparent zone around the bacterial colony was considered an indicator of a positive reaction for phosphate solubilization or potassium release. A yellow halo formed around the bacterial colony was considered an indicator of a positive reaction for siderophore production. The ratio of the halo diameter to the colony diameter was defined as the m_p_ value. Strains that grew well in nitrogen-free Ashby’s medium were recognized as nitrogen-fixing bacteria. The yields of IAA produced by bacteria cultured in precursor L-tryptophan medium were quantified using chloride perchloric acid reagent (FeCl_3_-HClO_4_) by colorimetric methods (Balasjin et al., 2022). ACC deaminase was induced on Dworkin & Foster (DF) salt minimal medium containing 3 mM ACC as the sole nitrogen source (Penrose and Glick, 2003). ACC deaminase activity was quantified by the amount of α-ketobutyrate produced by the cleavage of ACC. It was defined as the amount of α-ketobutyrate (μmol) produced by per mg of enzyme per hour. All traits were tested in triplicate and repeated three times. The *nifH* gene (PolF primer, 5’- TGCGAYCCSAARGCBGACTC-3’ and PolR primer, 5’- ATSGCCATCATYTCRCCGGA-3’) and *acdS* gene encoding ACC deaminase (acdSf3 primer, 5’-ATCGGCGGCATCCAGWSNAAYCANAC-3’ and acdSr4 primer, 5’ -GGCACGCCGCCCARRTGNRCRTA-3’) were amplified and sequenced (Pang et al., 2020).

### Morphological and molecular identification of isolates

Colony morphology and bacterium size of isolates were observed based on purified cultures on NA plates and Gram staining, respectively. The full-length 16S rRNA sequences of isolates were amplified using the following primers (27 forwards, 5’- AGAGTTTGATCCTGGCTCAG-3’ and 1492 reverse, 5’-GGTTACCTTGTTACGACTT-3’). The PCR amplification reaction (25 L) contained 2.5 μL of 10×*Ex Taq* Buffer (Mg^2+^ plus) (Takara, Beijing, China), 2 μL of dNTP mixture, 50 ng of the bacterial genomic DNA, 200 nM of each primer, 1.25 U of *Ex Taq* (Takara, Beijing, China) and 18.25 μL of H_2_O. The PCR amplification conditions were as follows: denaturation at 94°C for 2 min, followed by 35 cycles of 94°C for 30 s, 55°C for 30 s, 72°C for 1.5 min and final extension at 72°C for 5 min. After purification and sequencing, amplicons were analysed using the BLASTn algorithm in the NCBI GenBank database and then deposited in the GenBase database under the accession numbers presented in **Table S4**, which are publicly accessible at https://ngdc.cncb.ac.cn/genbase/. The phylogenetic tree was constructed using MEGA software (version 11) with the neighbour-joining method.

### Growth promotion assays on perennial rice

Healthy seeds of perennial rice (Geng/*japonica* cultivar PR23 and Xian/*indica* cultivar PR107) were surface-sterilized with 70% ethanol (v/v) for 1 min, followed by 30 min in 50% (v/v) commercial bleach with shaking at 180 rpm. Seeds were then washed 8-10 times with sterile distilled water and dried on autoclaved Whatman paper (3 mm) for 5 min. Then the surface sterilized seeds were soaked in 1×10^8^ cfu/mL strain suspension for 12 h, whereas the control seeds were soaked in LB medium. After germination in the dark for 1 d at 35°C, 9 seeds were planted in a pot filled with sterile conventional nutrient soil (weakly acidic soil contained enough nitrogen, phosphorus and potassium) and placed in a growth chamber under controlled conditions with 14 h daylight at 28°C and a 10 h dark period at 25°C. Each treatment was repeated with three pots in triplicate. At the 4-leaf seedling stage of 30 days after germination, chlorophyll (SPAD) and nitrogen content were measured using a TYS-4 N leaf metre (Jinkelida, Beijing, China). Seedling height, root length, fresh weight and dry weight were then determined at the seedling stage.

### Statistical analysis

Data are presented as the mean ± SD and were compared with SPSS software (version 20.0) using a one-way analysis of variance (ANOVA) according to Duncan’s multiple range test.

## Results

### Community composition of endophytic bacteria

**Figure 1:**
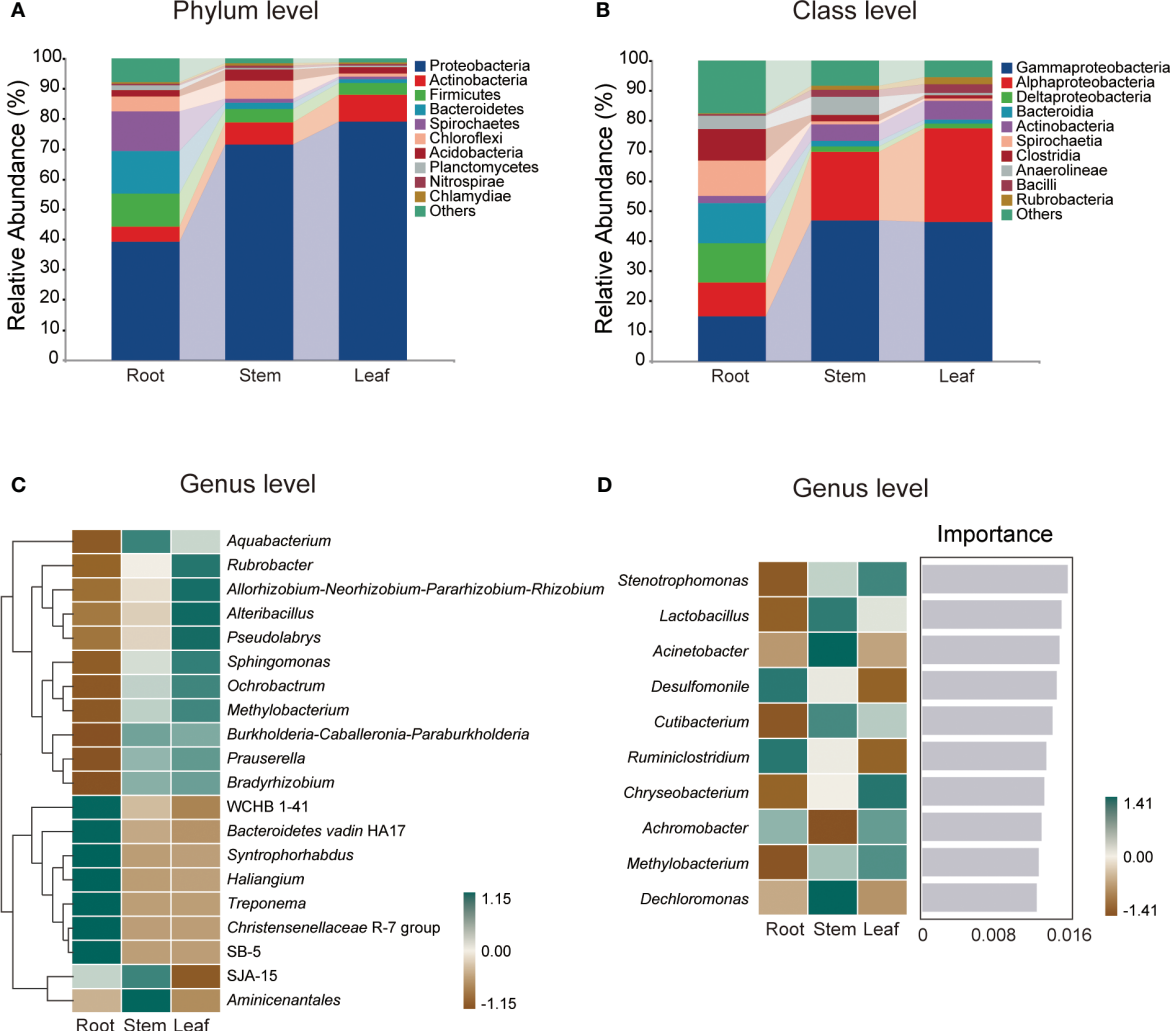
Relative abundances of the endophytic bacterial dominant phyla **(A)** and classes **(B)** in *Oryza officinalis*. The heatmap shows the top 20 relative abundances of the dominant endophytic bacterial genera in each tissue **(C)**. The importance of the top 10 dominant endophytic bacterial genera is identified based on random forest classification models and their relative abundance in each tissue **(D)**.

A total of 1,280,955 high-quality sequences were generated from 12 samples and assigned to 4,565 ASVs of root endophytic bacteria (REB), 850 ASVs of stem endophytic bacteria (SEB) and 719 ASVs of leaf endophytic bacteria (LEB) (**Figure S1A**). The number of ASVs, specific ASVs and taxa were highest in REB, followed by those in SEB, whereas the lowest numbers were observed in LEB (**Figures S1B, C**). Proteobacteria (39.30-79.12%) and Gammaproteobacteria (14.94-46.28%) were the dominant phylum and class, respectively, across all tissue endospheres of *O. officinalis* (**Figures 1A, B**). The average relative abundance of the top 10 dominant bacterial classes exceeded 1% (**Figure 1B**).

In addition, the relative abundance of the taxa at each taxonomic level exhibited considerable variation among the different tissue types. Among the top 20 relative abundances of genera, WCHB1-41, *Bacteroidetes_vadin* HA17, *Syntrophorhabdus*, *Haliangium*, *Treponema*, Christensenellaceae R-7 group and SB-5 were dominant in the root endosphere; *Aquabacterium*, SJA-15 and *Aminicenantales* were dominant in the stem endosphere; and *Rubrobacter*, *Allorhizobium-Neorhizobium-Pararhizobium-Rhizobium*, *Alteribacillus*, *Pseudolabrys*, *Sphingomonas*, *Ochrobactrum*, and *Methylobacterium* were dominant in the leaf endosphere (**Figure 1C**).

Based on random forest classification model analysis, 10 bacterial genera were predicted as biomarkers in the endospheres of *O. officinalis*, and their relative abundances also differed among tissue types (**Figure 1D**). For example, the relative abundances of *Desulfomonile* and *Ruminiclostridium* were highest in the REB; the relative abundances of *Lactobacillus*, *Acinetobacter*, *Cutibacterium* and *Dechloromonas* were highest in the SEB; and the relative abundances of *Stenotrophomonas*, *Chryseobacterium*, *Achromobacter* and *Methylobacterium* were highest in the LEB, suggesting that the bacterial endospheres of *O. officinalis* have tissue-specific biomarkers. However, no common taxa were found in all three compartments These results indicate that the differentiation and growth of *O. officinalis* tissues shape the discriminative endophytic bacterial communities.

### Diversity of endophytic bacterial communities

**Figure 2:**
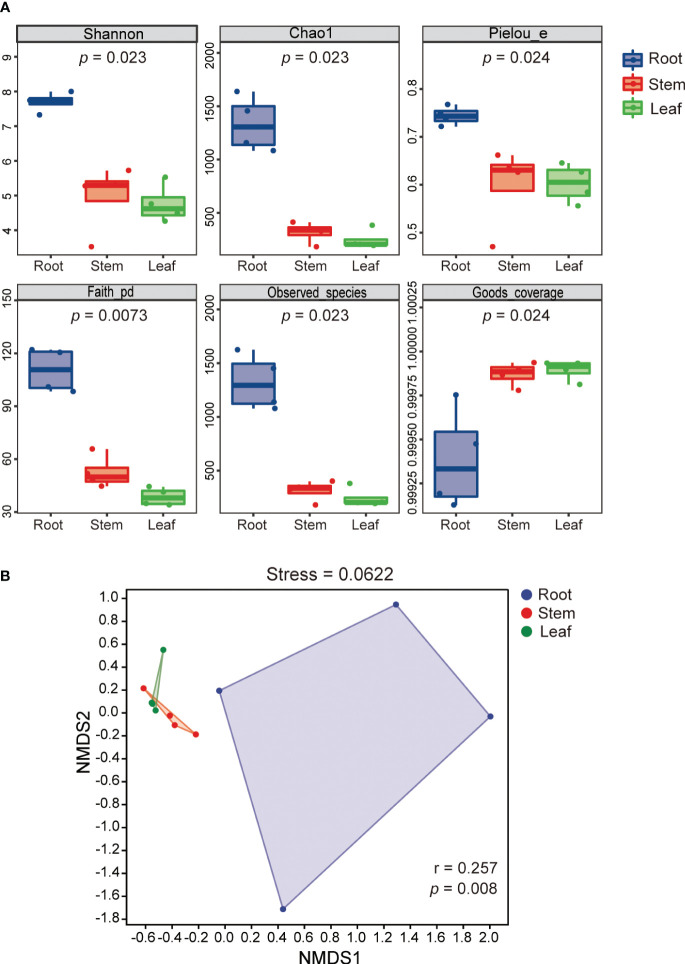
Shannon index (species diversity), Chao1 (species richness), Pielou index (species evenness), Faith-PD (phylogenetic diversity), observed species and Good’s coverage of endophytic bacterial communities in different tissues of *Oryza officinalis* are presented in box plots of alpha diversity. The *p* value of the Kruskal-Wallis test is shown under the alpha diversity index label **(A)**. Nonmetric multidimensional scaling plot (NMDS) analysis for endophytic bacterial communities associated with *O. officinalis* was estimated using Bray-Curtis distance and ANOSIM similarity tests **(B)**.

The alpha diversity indices (Shannon, Chao1, Pielou, Faith-PD and observed species) of REB were significantly higher than those of SEB and LEB (*p* < 0.05), indicating that species diversity, richness and evenness as well as the phylogenetic diversity of REB were highest across all tissue endospheres of *O. officinalis* (**Figure 2A**). The Good’s coverage index of the REB, SEB and LEB samples was greater than 99.9%, suggesting that the data were highly representative of the actual endophytic bacterial communities of *O. officinalis* (**Figure 2A**). Beta-diversity analysis using NMDS ordinations and ANOSIM also confirmed the significant differences among REB, SEB and LEB (*p* < 0.01) (**Figure 2B**). These results indicate that the roots of *O. officinalis* may harbor a distinct endophytic bacterial community structure with the greatest diversity, whereas the stems and leaves may share homologous endophytic bacterial community structures.

### Functional profiles and co-occurrence network of the endophytic bacterial community

**Figure 3:**
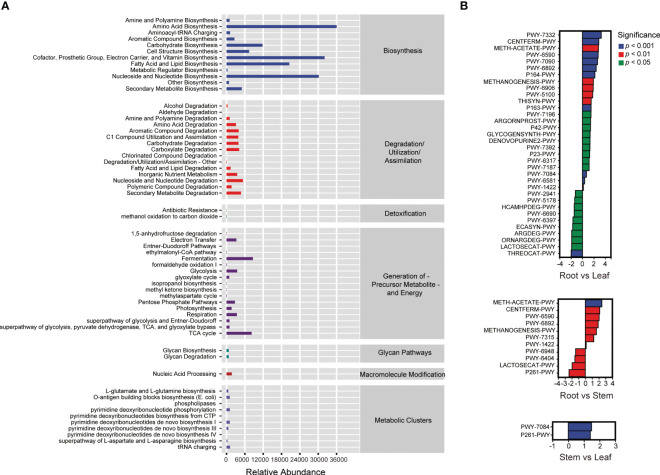
MetaCyc pathways of endophytic bacteria in *Oryza officinalis* **(A)** and the significantly differential MetaCyc pathways enriched in the root vs. leaf, root vs. stem and stem vs. leaf comparison groups **(B)**.

A total of 59 MetaCyc pathways were predicted in the endophytic bacterial communities of *O. officinalis* (**Figure 3A**). The relative abundance of ASVs involved in biosynthesis was highest in comparison with those involved in other metabolic pathways, suggesting that endophytic bacteria of *O. officinalis* may produce various metabolites to interact with their communities and host. Thirty-four, eleven and two significant differential MetaCyc pathways were enriched in the comparison groups of root vs. leaf, root vs. stem and stem vs. leaf, respectively, indicating that the root endosphere had more features and functionality of biosynthesis, degradation and biology; furthermore, the stem and leaf endospheres shared various common functions (**Figure 3B** and **Table S1**).

The endophytic bacterial co-occurrence network of *O. officinalis*, with an average degree of 64.81, had three putative modules that might be reassembled for these specific ecological functions (**Figure S2A**). A total of 178 ASVs constituted the network nodes among which 50 ASVs coacted in tight Module 1 and 74 and 54 ASVs coacted in loose Module 2 and Module 3, respectively (**Table S2**). The genera SJA-15, *Methylobacterium*, *Alteribacillus*, *Burkholderia*-*Caballeronia*-*Paraburkholderia*, *Ochrobactrum*, *Aquabacterium*, *Prauserella*, WCHB1-41, *Bradyrhizobium*, and *Bacteroidetes vadin* HA17 were the top nodes in this network (**Figure S2B**). There was no obvious difference in the number of nodes and edges and the average degree among subnetworks for REB, SEB and LEB, which is perhaps related to the small sample size of each subnetwork (**Figure S2C**).

### *In vitro* plant growth-promoting abilities

**Figure 4:**
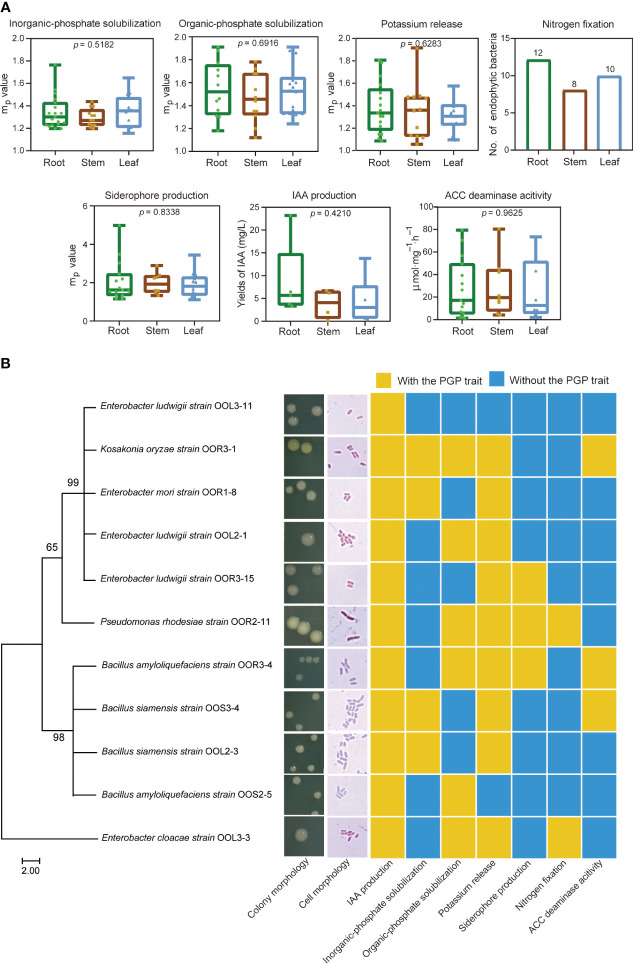
Plant growth-promoting bacteria isolated from *O. officinalis* were determined *in vitro.* Each PGP ability value of the strains isolated from the root, stem and leaf tissues are represented by green, brown and blue dots, respectively **(A)**. Phylogenetic tree, morphological characteristics and PGP traits of 11 strains selected for inoculation of perennial rice **(B)**.

A total of 99 endophytic bacterial strains were isolated from the root, stem and leaf tissues of *O. officinalis*, including 42 REB, 26 SEB and 31 LEB (**Table S3**). Among them, 96 endophytic bacterial strains had at least one PGP trait of phosphate solubilization, potassium release, nitrogen fixation, ACC deaminase activity, or siderophore/IAA production (**Figure 4**, **Figure S3** and **Table S3**). In addition, *nifH* and *acdS* genes were detected in 19 and 34 strains with nitrogen fixating abilities and ACC deaminase activity, respectively, indicating that they had the genetic potential for N_2_ fixation and ethylene regulation (**Figure S3**). Thus, these strains were preliminarily characterized as PGPB. The number of PGPB isolated from root tissues was greater than that isolated from stem and leaf tissues. However, their PGP abilities *in vitro* were not significantly different among the REB, SEB and LEB (**Figure 4A**). These results suggest that *O. officinalis* may assemble a large number of endophytic bacteria with PGP traits in each tissue compartment to aid in the acquisition of nutrients from the environment, phytohormone production and stress tolerance.

Eleven strains were selected for further study of their effects on perennial rice based on their IAA production abilities (yields >3 mg/L) (**Figure 4B** and **Table S3**). Based on morphological observations and full-length 16S rRNA sequence analysis, these strains were identified as *Enterobacter mori* (strain OOR1-8), *Enterobacter ludwigii* (strains OOR3-15, OOL2-1 and OOL3-11), *Enterobacter cloacae* (strain OOL3-3), *Bacillus amyloliquefaciens* (strains OOR3-4 and OOS2-5), *Bacillus siamensis* (strains OOS3-4 and OOL2-3), *Pseudomonas rhodesiae* (strain OOR2-11), and *Kosakonia oryzae* (strains OOR3-1) (**Figure 4B** and **Table S4**).

### Plant growth-promoting effects on perennial rice

**Figure 5:**
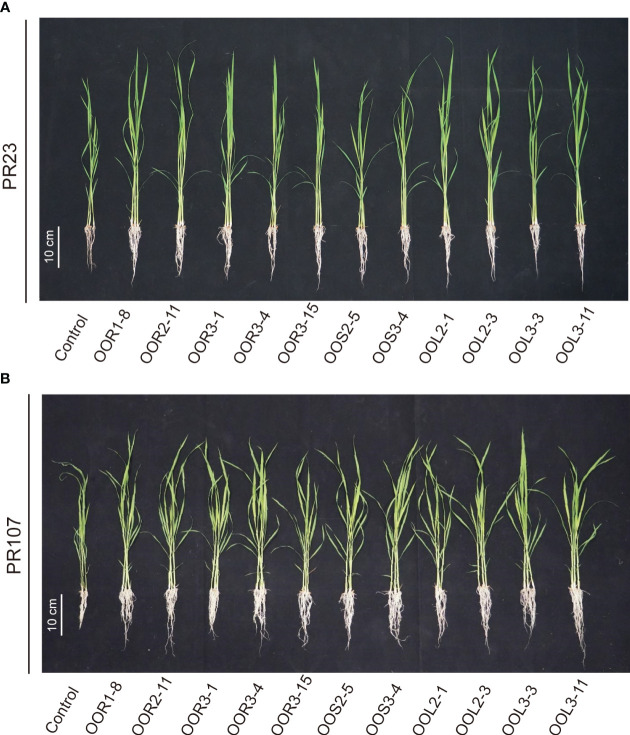
Effects of 11 bacteria strains isolated from *Oryza officinalis* on perennial rice growth. PR23 is a *japonica* subspecies **(A)**, and PR107 is an *indica* subspecies **(B)**.

Seeds from the perennial rice varieties PR23 (*japonica* subspecies) and PR107 (*indica* subspecies) were inoculated individually with each of the eleven strains. Generally, inoculated PR23 and PR107 grew faster and stronger than the noninoculated control plants (**Figure 5**). Except for *B. amyloliquefaciens* OOS2-5, most strains significantly promoted the seedling height, fresh weight, chlorophyll and nitrogen content of PR23, and *E*. *mori* OOL3-11 and *E. ludwigii* OOR3-15, OOR1-8 and OOL2-1 also promoted the root length of PR23 (**Table 1**). All strains significantly promoted the fresh weight and chlorophyll content of PR107 and most promoted the root length and nitrogen content of PR107. Only *P*. *rhodesiae* OOR2-11 and *B. siamensis* OOL2-3 promoted the seedling height of PR107 (**Table 2**). These results suggest that *E. mori*, *E. ludwigii*, *E. cloacae*, *B. amyloliquefaciens*, *B. siamensis*, *P. rhodesiae* and *K. oryzae* with IAA production and other PGP traits could promote perennial rice seedling development and enhance their biomass accumulation, chlorophyll content, and nitrogen uptake.

**Table 1:** Growth-promoting effects of 11 endophytic bacterial strains isolated from *Oryza officinalis* on perennial rice PR23.

Inoculated strain	Seedling height (cm)	Root length (cm)	Fresh weight (g)	Dry weight (g)	Dry weight rate of shoot/root	Chlorophyll content (SPAD)	Nitrogen content (g/kg)
Noninoculated control PR23	32.62 ± 0.66c	10.02 ± 0.36b	0.41 ± 0.06c	0.11 ± 0.01c	5.04 ± 0.24b	28.08 ± 1.18d	11.42 ± 0.43c
*Enterobacter mori* OOR1-8	36.89 ± 2.06a	11.35 ± 1.47a	0.58 ± 0.05a	0.13 ± 0.01ab	5.01 ± 0.65b	33.46 ± 0.98a	13.18 ± 0.33a
*Pseudomonas rhodesiae* OOR2-11	37.06 ± 1.51a	10.75 ± 0.87ab	0.58 ± 0.09a	0.13 ± 0.01ab	5.13 ± 0.35b	32.33 ± 0.97ab	13.02 ± 0.50a
*Kosakonia oryzae* OOR3-1	36.31 ± 3.32ab	10.69 ± 0.87ab	0.55 ± 0.05a	0.12 ± 0.01bc	5.08 ± 0.64b	32.20 ± 0.99ab	12.38 ± 0.38b
*Bacillus amyloliquefaciens* OOR3-4	35.96 ± 3.31b	10.71 ± 1.49ab	0.46 ± 0.12b	0.11 ± 0.02c	5.15 ± 0.61ab	31.92 ± 2.02ab	12.34 ± 0.62b
*Enterobacter ludwigii* OOR3-15	35.74 ± 0.86b	11.48 ± 1.88a	0.51 ± 0.04ab	0.12 ± 0.01bc	4.97 ± 0.31bc	30.78 ± 1.80bc	12.28 ± 0.49b
*Bacillus amyloliquefaciens* OOS2-5	32.88 ± 0.46c	11.13 ± 0.75ab	0.44 ± 0.04bc	0.11 ± 0.01c	4.92 ± 0.21c	28.94 ± 1.70cd	11.87 ± 0.54bc
*Bacillus siamensis* OOS3-4	36.25 ± 3.01ab	10.21 ± 1.44b	0.47 ± 0.06b	0.12 ± 0.02bc	5.07 ± 0.53b	30.94 ± 0.75bc	12.69 ± 0.40ab
*Enterobacter ludwigii* OOL2-1	37.84 ± 0.68a	11.36 ± 0.76a	0.52 ± 0.01ab	0.14 ± 0.01a	5.05 ± 0.24b	31.62 ± 0.49b	13.43 ± 0.14a
*Bacillus siamensis* OOL2-3	37.29 ± 0.88a	10.34 ± 0.81b	0.53 ± 0.06ab	0.13 ± 001ab	5.21 ± 0.30a	32.03 ± 0.28ab	12.99 ± 0.10a
*Enterobacter cloacae* OOL3-3	35.65 ± 2.37b	10.08 ± 1.06b	0.44 ± 0.07bc	0.11 ± 0.01bc	5.12 ± 0.58b	29.76 ± 2.94c	11.98 ± 0.66bc
*Enterobacter ludwigii* OOL3-11	36.01 ± 1.50b	11.47 ± 0.62a	0.52 ± 0.03ab	0.14 ± 0.01a	4.95 ± 0.46c	31.76 ± 0.73b	12.76 ± 0.25ab

Mean ± SD with different letters in the same column are significantly different (p < 0.05) based on Duncan’s multiple mean comparison test.

**Table 2:** Growth-promoting effects of 11 endophytic bacterial strains isolated from *Oryza officinalis* on perennial rice PR107.

Inoculated strain	Shoot height (cm)	Root length (cm)	Fresh weight (g)	Dry weight (g)	Dry weight rate of shoot/root	Chlorophyll content (SPAD)	Nitrogen content (g/kg)
Noninoculated control PR107	35.13 ± 0.78b	9.55 ± 0.34c	0.44 ± 0.05d	0.13 ± 0.01c	5.23 ± 0.34a	28.82 ± 1.86c	11.92 ± 0.37b
*Enterobacter mori* OOR1-8	35.73 ± 2.45ab	11.84 ± 0.39ab	0.65 ± 0.04a	0.16 ± 0.02ab	4.97 ± 0.62c	32.50 ± 1.63a	13.05 ± 0.58a
*Pseudomonas rhodesiae* OOR2-11	36.37 ± 1.40a	12.32 ± 0.59a	0.67 ± 0.05a	0.17 ± 0.01ab	4.95 ± 0.41c	32.75 ± 1.04a	12.95 ± 0.31a
*Kosakonia oryzae* OOR3-1	35.88 ± 1.01ab	10.68 ± 0.66bc	0.57 ± 0.07b	0.15 ± 0.01b	5.18 ± 0.35ab	31.35 ± 0.89ab	12.52 ± 0.28ab
*Bacillus amyloliquefaciens* OOR3-4	35.75 ± 0.64ab	11.82 ± 0.29ab	0.65 ± 0.06a	0.16 ± 0.01ab	5.10 ± 0.28b	32.27 ± 0.45a	13.18 ± 0.16a
*Enterobacter ludwigii* OOR3-15	35.34 ± 0.90b	11.68 ± 0.83ab	0.52 ± 0.04c	0.15 ± 0.01b	5.11 ± 0.25b	30.28 ± 1.63b	12.45 ± 0.52ab
*Bacillus amyloliquefaciens* OOS2-5	35.83 ± 0.91ab	11.77 ± 0.71ab	0.61 ± 0.06ab	0.16 ± 0.01ab	5.11 ± 0.26b	32.65 ± 0.76a	12.98 ± 0.23a
*Bacillus siamensis* OOS3-4	35.75 ± 1.67ab	11.63 ± 0.66ab	0.65 ± 0.05a	0.16 ± 0.01ab	5.13 ± 0.47b	31.50 ± 1.3ab	12.88 ± 0.47a
*Enterobacter ludwigii* OOL2-1	35.56 ± 0.86ab	11.60 ± 0.53ab	0.55 ± 0.05bc	0.15 ± 0.01b	5.12 ± 0.26b	31.22 ± 0.45ab	12.83 ± 0.14a
*Bacillus siamensis* OOL2-3	36.33 ± 0.88a	11.95 ± 1.39ab	0.58 ± 0.05b	0.15 ± 0.01b	5.11 ± 0.18b	31.58 ± 0.73ab	12.03 ± 0.33b
*Enterobacter cloacae* OOL3-3	35.87 ± 1.03ab	11.27 ± 0.74b	0.61 ± 0.04ab	0.16 ± 0.01ab	5.11 ± 0.23b	32.52 ± 0.75a	13.23 ± 0.24a
*Enterobacter ludwigii* OOL3-11	35.67 ± 0.95ab	12.21 ± 0.81a	0.62 ± 0.04ab	0.16 ± 0.01ab	4.97 ± 0.21c	32.58 ± 0.69a	13.27 ± 0.20a

Mean ± SD with different letters in the same column are significantly different (p < 0.05) based on Duncan’s multiple mean comparison test.

## Discussion

### Endophytic bacterial communities in rice strongly vary by host genotype

Wild rice, including 22 species of genome types (AA, BB, CC, EE, FF, GG, BBCC, CCDD, HHJJ and KKLL), possesses much higher genetic diversity than domesticated rice (*O. sativa*) (Kajiya-Kanegae et al., 2021). Their genotypes mainly determine the assembly of plant-associated microbiome, suggesting that wild rice recruits more powerful host-associated microbiomes to regulate environmental adaptation than domesticated rice (Yin et al., 2023). Rhizosphere and phyllosphere microbial communities have been characterized in *O. rufipogon* and *O. longistaminata*, while the endophytic microbiome has rarely been reported in most wild rice relatives (Peng et al., 2021; Xie et al., 2021; Chang et al., 2022; Xu et al., 2022). Our study provided insight into the endophytic bacterial community in *O. officinalis*. Compared to previous studies, the endophytic bacterial composition of wild rice and cultivated rice is consistent at the phylum level and class level regardless of genetic and ecological distance. Proteobacteria and Gammaproteobacteria are typically the dominant phylum and class in rice, respectively (Moronta-Barrios et al., 2018; Kim and Lee, 2020; Lin et al., 2020; Chu et al., 2021; Kunda et al., 2021; Peng et al., 2021).

Endophytic bacterial microbiomes exhibit high variation in genus composition and abundance patterns among different rice species and varieties, resulting in different host-specific biomarkers. For instance, *Desulfomonile*, *Lactobacillus*, *Stenotrophomonas*, etc., are biomarkers of the endophytic bacterial microbiome of *O. officinalis*, compared with *Methylobacteriaceae* and *Streptococcus* in African wild rice (*O. longistaminata*) and *Pseudomonas* and *Ralstonia* in cultivated rice (Chu et al., 2021; Peng et al., 2021).

The microbial interaction network with high correlations between microbial groups maintains a stable and sustainable ecological network to respond to environmental changes, which are directly beneficial for the host (Coyte et al., 2015). The key set of microbial species in the cooperative network could play a critical role in driving the formation of communities and promoting microecological stability. *O. officinalis* uses *Methylobacterium*, *Alteribacillus*, *Burkholderia*-*Caballeronia*-*Paraburkholderia*, *Ochrobactrum*, *Aquabacterium*, etc., to establish linkages between endophytic bacteria. The endophytic bacterial network structure of *O. officinalis* showed a different connectivity pattern than those of *O. longistaminata* and cultivated rice (Chu et al., 2021; Peng et al., 2021; Singha et al., 2021; Xie et al., 2021). Thus, rice genotype is the critical influencing factor of their endophytic bacterial community structure and interaction.

### Endophytic bacteria of different taxa and ecological functions colonize the tissue compartments of *Oryza officinalis*

Due to the differentiation of structure, function and nutrient environment in tissues, rice plants shape distinctive ecological niches for endophytes in the different compartments. The root endosphere has received more attention than the stem and leaf endospheres in most previous studies because of its importance for interactions between roots and soil (Beckers et al., 2017; Yu and Hochholdinger, 2018; Carrion et al., 2019; Kim and Lee, 2020; Sun et al., 2021). The frequent exchange of microbiota between the root endosphere and rhizosphere results in a larger and more effective endophytic microbiota being recruited by rice roots than by aboveground compartments.

In this study, the bacterial composition, abundance, diversity, and functional profile of the root endosphere in *O. officinalis* were significantly different from those of the leaf and stem endospheres. Similar results were found in the endospheres of *O. longistaminata*, black rice and other plants (Sun et al., 2020; Kim and Lee, 2021; Peng et al., 2021; Singha et al., 2021; Lin et al., 2022; Sun et al., 2022). *Desulfomonile* and *Ruminiclostridium*, with the capability of nitrogen fixation and cellulose degradation (Ju et al., 2007; Kampik et al., 2020), are REB-specific biomarkers of *O. officinalis*. *Lactobacillus*, *Acinetobacter*, and *Dechloromonas* are SEB-specific biomarkers of *O. officinalis* and are involved in inhibition against phytopathogenic bacteria, nitrogen removal, biodegradation and iron oxidation (Chakraborty and Picardal, 2013; Silambarasan and Vangnai, 2016; Tsuda et al., 2016; Ke et al., 2022). *Stenotrophomonas*, *Chryseobacterium*, *Achromobacter* and *Methylobacterium*, as LEB-specific biomarkers of *O. officinalis*, can promote plant growth and nutrient uptake, suppress plant pathogens and reduce arsenic accumulation in rice grains (Suckstorff and Berg, 2003; Kim et al., 2010; Sang and Kim, 2012; Wang et al., 2020a). These results indicated that endophytic bacteria with PGP potential assemble and colonize different compartments of *O. officinalis*, whose functions may evolve compatibly with the functions of the host tissues. Moreover, tissue-specific endophytic bacterial biomarkers are firmly controlled by rice genotype. For instance, *Rhizobium*, AB2 and *Leptonema* are LEB-specific biomarkers of *O. longistaminata*; *Methylobacterium* and *Nevskia* are SEB-specific biomarkers; while *Enhydrobacter*, YS2 and *Roseburia* are rhizome-specific biomarkers (Peng et al., 2021), which are completely different from *O. officinalis*.

### Endophytic bacteria of *Oryza officinalis* are the resource pool of plant-growth-promoting bacteria

Many endophytic bacteria with PGP traits have been isolated from wild rice species such as *O. alta*, *O. brachyantha*, *O. eichingeri*, *O. latifolia*, *O. longiglumis*, *O. meridionalis*, *O. minuta*, *O. officinalis*, *O. punctata*, *O. ridley and O. rufipogon* (Borah et al., 2021; Zhang et al., 2021; Liu et al., 2022b). Only a few species have been applied to cultivated rice, and even fewer have been applied to perennial rice. *Microbacterium* strain B24 with cell wall degradation enzyme activities and *Herbaspirillum* strain B501 with the ability to fix nitrogen in plants were isolated and functionally identified from *O. officinalis* (Elbeltagy et al., 2000; Elbeltagy et al., 2001). In this study, 96.97% of endophytic bacterial strains isolated from *O. officinalis* had at least one PGP trait of phosphate solubilization, potassium release, nitrogen fixation, ACC deaminase, or siderophore/IAA production. The PGPB isolation efficiency is remarkably higher from endophytic bacteria of *O. officinalis* than from those of *O. rufipogon* (28.48%) (Zhang et al., 2021). Plant genomic diversity positively regulates the composition and diversity of phytomicrobiome (Sutherland et al., 2022; Zhang et al., 2023). Previous studies showed that the *O. officinalis* genome (average 10.26 Gbp) was larger than *O. rufipogon* genome (average 6.83 Gbp), indicating that *O. officinalis* has higher genetic diversity than *O. rufipogon* (Kajiya-Kanegae et al., 2021). Thus, *O. officinalis* might also have higher microbiome diversity than *O. rufipogon*, indicating more endophytic bacteria with the abilities of stress resistance and environmental adaptability in *O. officinalis*.

Colonization of PGPB in the rhizosphere and phyllosphere is a necessary prerequisite for their successful application under field conditions (Rilling et al., 2019). IAA production activity is the first useful trait for selecting promising endophytic bacteria with more PGP potential and a high colonization rate in rice plants (Etesami et al., 2015). Eleven strains identified as *Enterobacter*, *Bacillus*, *Pseudomonas* and *Kosakonia* species were selected for inoculation of perennial rice based on their IAA yields ranging from 3.02 to 23.2 mg/L. *Enterobacter*, *Bacillus* and *Pseudomonas* were detected from three tissues using 16S rRNA sequencing, but *Kosakonia* was not captured. The possible reason is either the extremely low abundance of *Kosakonia* species in *O. officinalis* or the low amplification efficiency for these species using 16S rRNA (V5-V7) sequence primers.

Using the method of soaking seeds, all taxa encourage perennial rice to form a strong root system, stimulate biomass accumulation, and increase chlorophyll content and nitrogen uptake, which are promising features of biofertilizers specifically targeted at the successive planting of perennial rice. Therefore, IAA trait *in vitro* is effective and time-saving for screening PGPB for perennial rice. Moreover, several *Enterobacter*, *Bacillus*, *Pseudomonas* and *Kosakonia* species have been reported to have potential for growth promotion and stress tolerance in rice (Sherpa et al., 2021; Raturi et al., 2022; Shukla et al., 2023; Yang et al., 2023). Interestingly, *E. ludwigii* OOL3-11 with only IAA production activity had a positive effect on perennial rice seedling growth, which was comparable with those strains with more PGP traits. This is probably because the PGP effects of those strains with more PGP traits are limited under normal perennial rice growth conditions. However, they may exert their PGP effects (e.g., ACC deaminase and siderophore production) sufficiently under nutrient deficiency and stress conditions (Sharma and Johri, 2003; Moon and Ali, 2022).

### *Bacillus amyloliquefaciens* OOS2-5 isolated from *Oryza officinalis* promotes perennial rice growth in a host genotype-dependent manner

*B. amyloliquefaciens* is a commercially successful biocontrol agent that can also enhance the yield of crop plants (Chowdhury et al., 2015). Interestingly, *B*. *amyloliquefaciens* OOS2-5 with the PGP traits of IAA production and organic-phosphate solubilization promotes PR107 (*indica* subspecies) growth but does not affect PR23 (*japonica* subspecies). The endophytic colonization and PGP effect of this strain has some differences in the *japonica* and *indica* subspecies. However, *B*. *amyloliquefaciens* OOR3-4 with more PGP traits of IAA production, organic-phosphate solubilization, potassium release, siderophore production and ACC deaminase activity can promote the growth of both perennial rice varieties. It is suggested that other PGP traits of *B*. *amyloliquefaciens* isolates are also important for endophytic colonization and the in-plant promoting effect on different hosts.

Four bacterial strains, *Pseudomonas mosselii*, *Microvirga*, *Paenibacillus rigui*, and *P. graminis* isolated from Asian rice species (*O. sativa*) showed a rice genotype-dependent manner of rice growth promotion (Balasjin et al., 2022), which is similar to *B*. *amyloliquefaciens* OOS2-5. These results also reveal that endophytic bacterial behavior with regard to rice growth is affected by rice genotype, which cannot be predicted by bacterial taxa and their PGP traits *in vitro*. It also reminds us that the application of microbial fertilizers for different perennial rice varieties needs to consider the bacterial isolate-specific and rice genotype-specific PGP effects.

## Conclusions

The composition, diversity and function of endophytic bacterial communities in the root, stem and leaf tissues of *O*. *officinalis* were first characterized in this work, revealing the tissue-specific endophytic bacterial microbiomes in *O*. *officinalis*. A number of endophytic bacteria with PGP traits were isolated from *O*. *officinalis*. Among them, 11 bacterial strains belonging to the *Enterobacter*, *Bacillus*, *Pseudomonas* and *Kosakonia* species promoted the growth of perennial rice seedlings and have utilization potential as biofertilizers for the sustained productivity of perennial rice. The stable and long-lasting PGP effects on perennial rice should be further evaluated in the field. Moreover, it is worth considerable research on the mechanism of interaction between these strains and different perennial rice varieties, which will make good use of bacteria to improve the agronomic traits of perennial rice.

## Data availability statement

The datasets presented in this study can be found in online repositories. The names of the repository/repositories and accession number(s) can be found in the article/**Supplementary Material**.

## Author contributions

SQ conceived and designed the research. QT and YG conducted the experiments and set up the data under SQ guidance. YG, SL and DK isolated and identified the endophytic bacteria. QT and MJ performed the bacterial phenotyping analysis. ZX, RT and LW performed in-plant phenotyping analysis under LH and FH guidance. FH provided rice materials. SQ and LH wrote the manuscript.
