# Supplementary material for: Endophytic bacterial communities in wild rice (Oryza officinalis) and their plant growth-promoting effects on perennial rice
Source: Front Plant Sci. 2023 Aug 14;14:1184489. doi: 10.3389/fpls.2023.1184489 (PMC10461003; doi:10.3389/fpls.2023.1184489)
Supplement: Supplementary file 1 [file DataSheet_1.zip › Supplementary Figures.pdf]

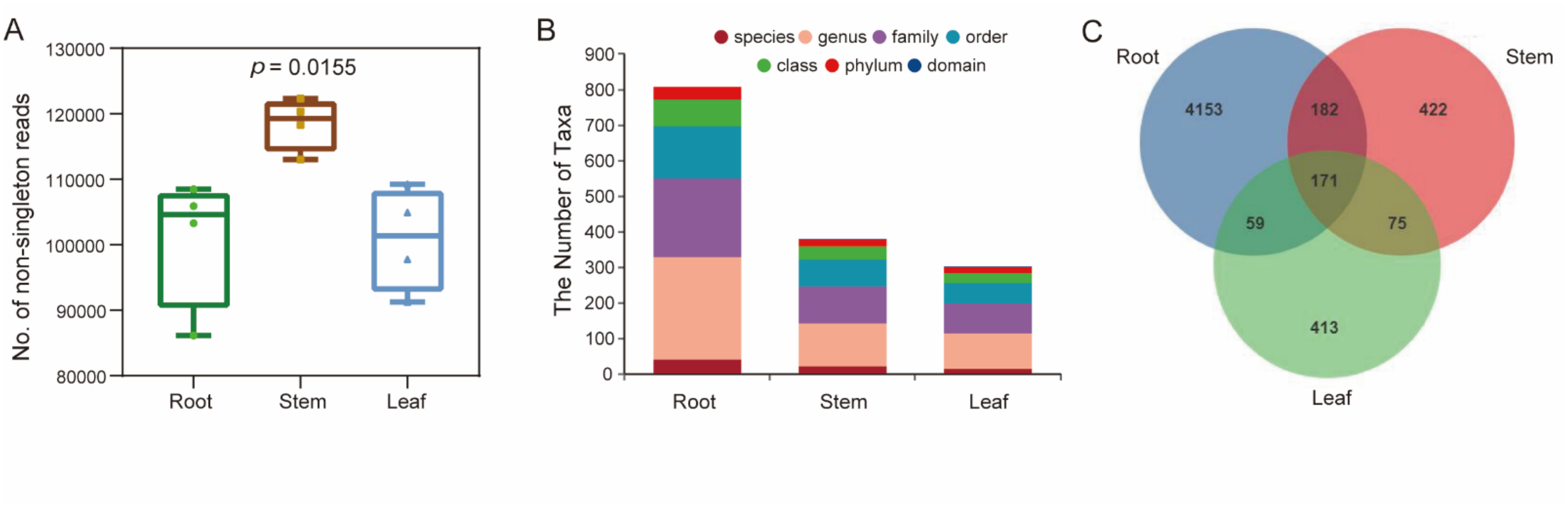

Fig. S1 Number of sequences with nonsingleton sequences and Taxa obtained using 16S rRNA gene Illumina sequencing technology (A) and common (B) and tissue-specific (C) endophytic bacterial ASVs in *Oryza officinalis*.

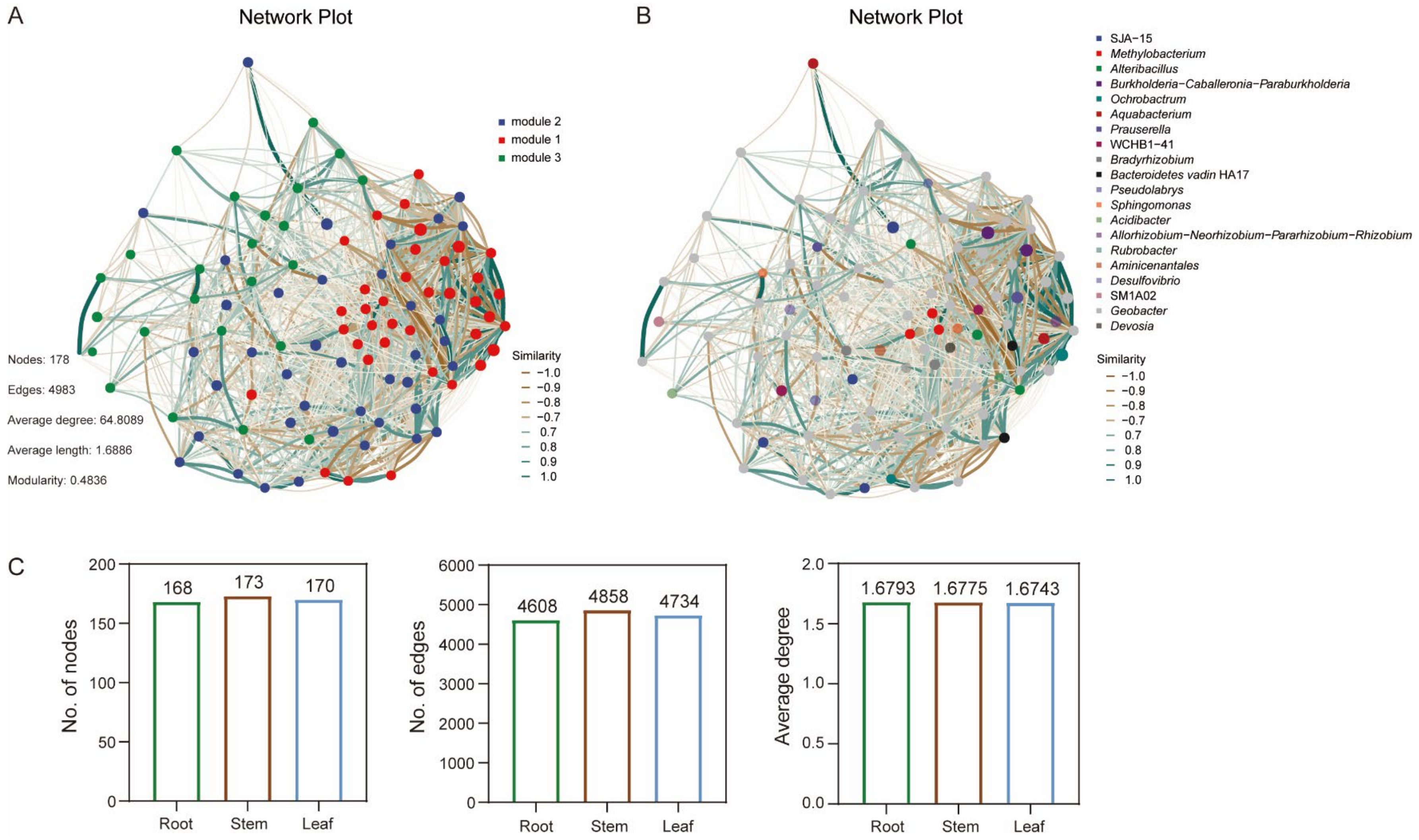

Fig. S2 Co-occurrence network of endophytic bacterial networks in *Oryza officinalis* using functional module analysis (A) and dominant bacterial genera analysis (B). Network properties of root, stem and leaf endospheres (C). Modules 1, 2 and 3 within the networks are shown in red, blue and green, respectively. Nodes represent the top 10 dominant genera of endophytic bacteria. The positive (shown in green) or negative correlation edge (shown in brown) between the two connected nodes.

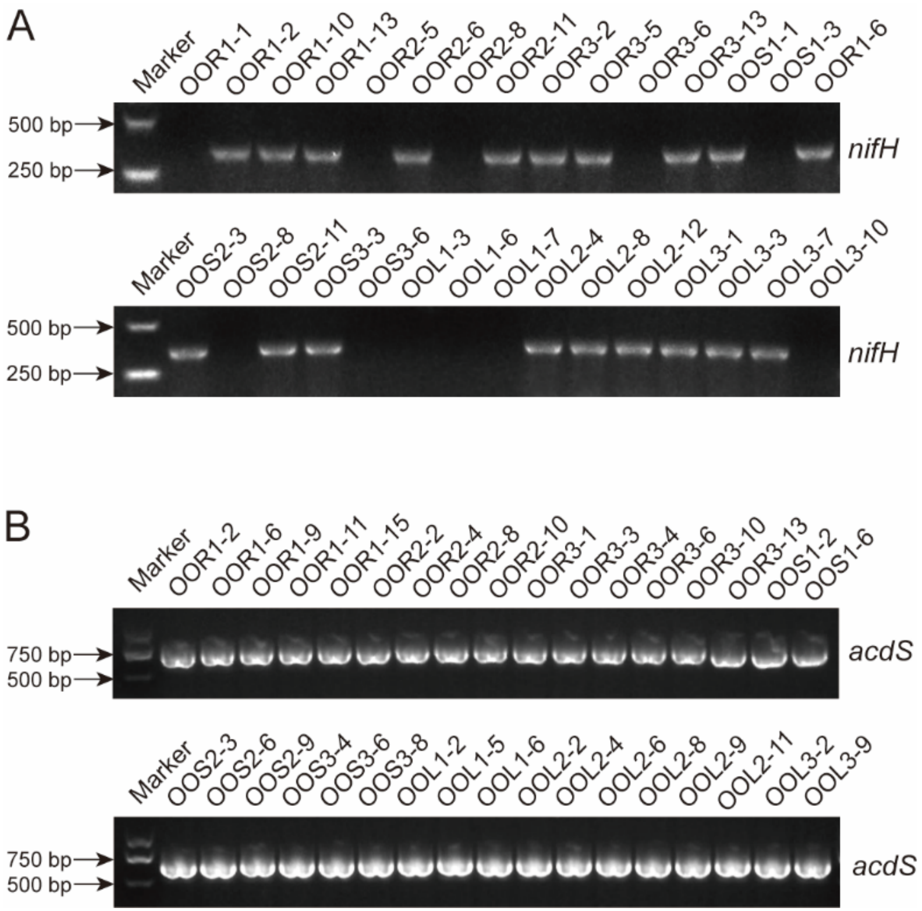

Fig. S3 *nifH* genes involved in nitrogen fixation (A) and *acds* genes encoding ACC deaminase (B) were amplified from strains isolated from *Oryza officinalis*.
